# Regression of bilateral persistent primitive ophthalmic artery: a case report

**DOI:** 10.1007/s00276-024-03522-7

**Published:** 2024-12-26

**Authors:** Issei Takeuchi, Takashi Izumi, Masahiro Nishihori, Shunsaku Goto, Taketo Hanyu, Keita Suzuki, Kai Takayanagi, Yuichi Kawasaki, Ryuta Saito

**Affiliations:** Department of Neurosurgery, Nagoya University of Graduate School of Medicine, Tsurumai-Cho 65, Showa-Ku, Nagoya, Aichi Japan

**Keywords:** Magnetic resonance angiography, Ophthalmic artery, Primitive ophthalmic artery, Ventral ophthalmic artery, Dorsal ophthalmic artery, Persistent artery, Regression

## Abstract

**Purpose:**

We report a case of regression of a 2-year-old girl with bilateral primitive ophthalmic arteries (POAs).

**Case report:**

The patient presented with a headache and had no visual impairment or visual field abnormalities. Magnetic resonance angiography (MRA) revealed arteries originating bilaterally from the cavernous internal carotid artery segments, diagnosed as persistent POAs.

**Conclusion:**

Six months later, MRA revealed decreased origin delineation and five years later, MRA revealed bilateral origin disappearance from the orbit to the periphery.

## Introduction

The ophthalmic artery (OA) is formed by the anastomosis and regression of several primary arteries, and several variations exist in its origin and anastomosis with the surrounding arteries [1, 2, 5–7, 9, 10]. Uchino et al. evaluated 1652 ophthalmic arteries in 826 cases by magnetic resonance angiography (MRA) and reported 24 cases (1.45%) of OA branching from the middle meningeal artery (MMA) and 7 cases (0.42%) of OA branching from the cavernous segment [10]. Other rare variants include the anterior cerebral and posterior communicating arteries, internal carotid artery (ICA) bifurcation, OA branching from the basilar artery, and OA originating from the MMA [2, 5–7, 9].

The natural regression of bilateral persistent ophthalmic arteries observed in this case, to the best of our knowledge, has not been previously described in the literature. Thus, we present this case with an embryological perspective on the ophthalmic artery.

## Case report

A 2-year-old girl presented with a headache, but she had no symptoms of visual field abnormality or disturbances. She was referred to a nearby pediatric hospital. Axial image of time-of-flight magnetic resonance angiography (MRA) revealed abnormal vascular shadows in the cavernous segments on both sides, leading to a referral to our hospital for further evaluation. She had no symptoms of visual field abnormality or visual field disturbances, only headache.

**Fig. 1:**
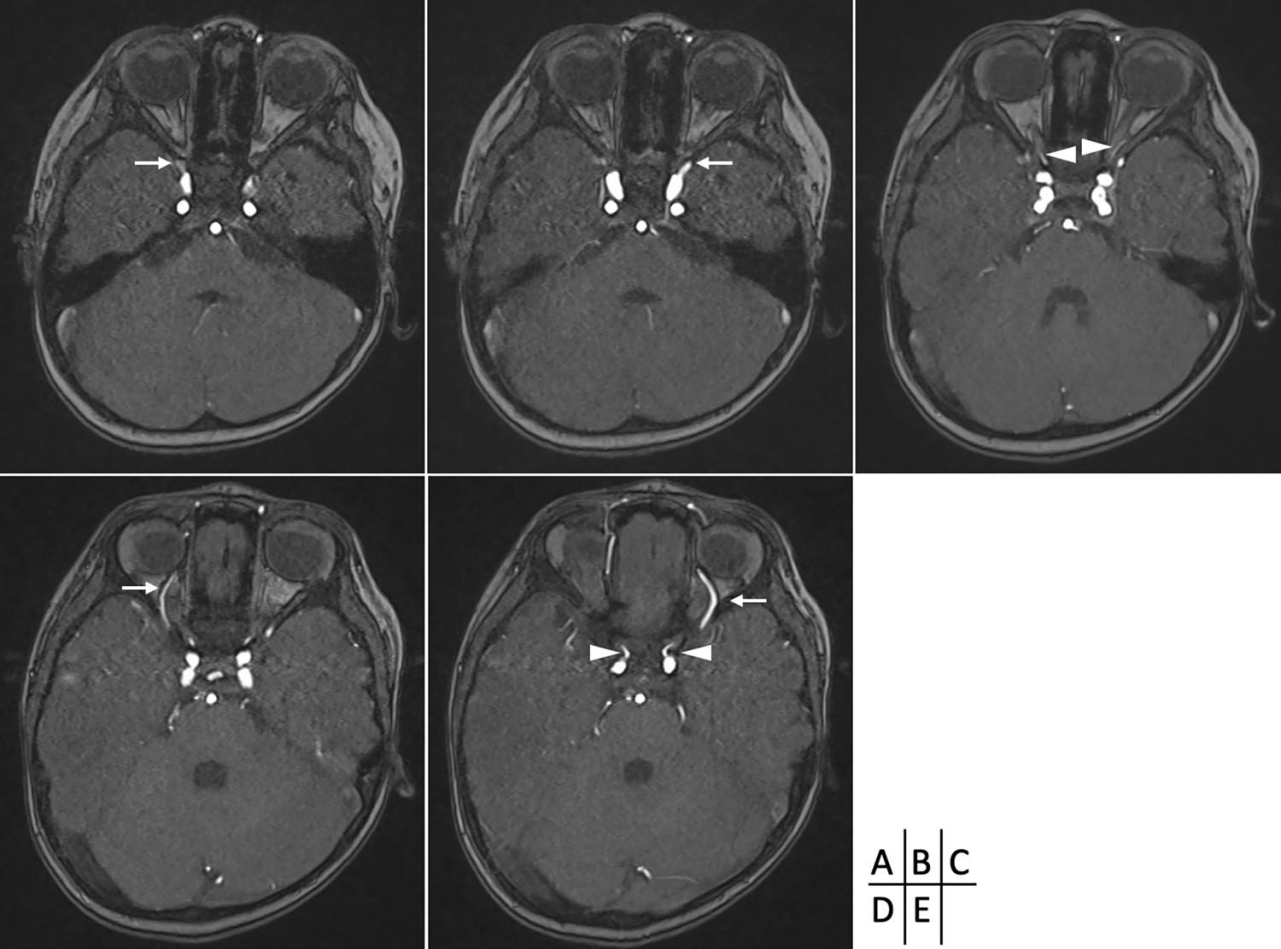
Time-of-flight of MRA showed the bilateral primitive ophthalmic arteries (POAs) observed originating from the cavernous internal carotid artery (ICA) segment. **A**, **B** They passed through the superior orbital fissure and extended toward the area near the nasal root. **C**, **D**, **E** Ophthalmic arteries (OA) were also observed originating from the clinoid ICA segment. **E** Both of these ran through the optic canal. **C** The arrows indicate the POA. The arrowheads indicate the OA

MRA revealed arteries originating from the clinoid and cavernous segments of the ICA on both sides. The former were within the optic canal and considered ophthalmic arteries. While, the latter passed through the superior orbital fissure, running over and medial to the eyeball with their terminals directed towards the area near the nasal root, observed bilaterally. The patient was diagnosed with the persistence of bilateral primitive ophthalmic arteries (POAs) (Fig. 1). No arteriovenous shunts or other lesions were observed in the facial area.

**Fig. 2:**
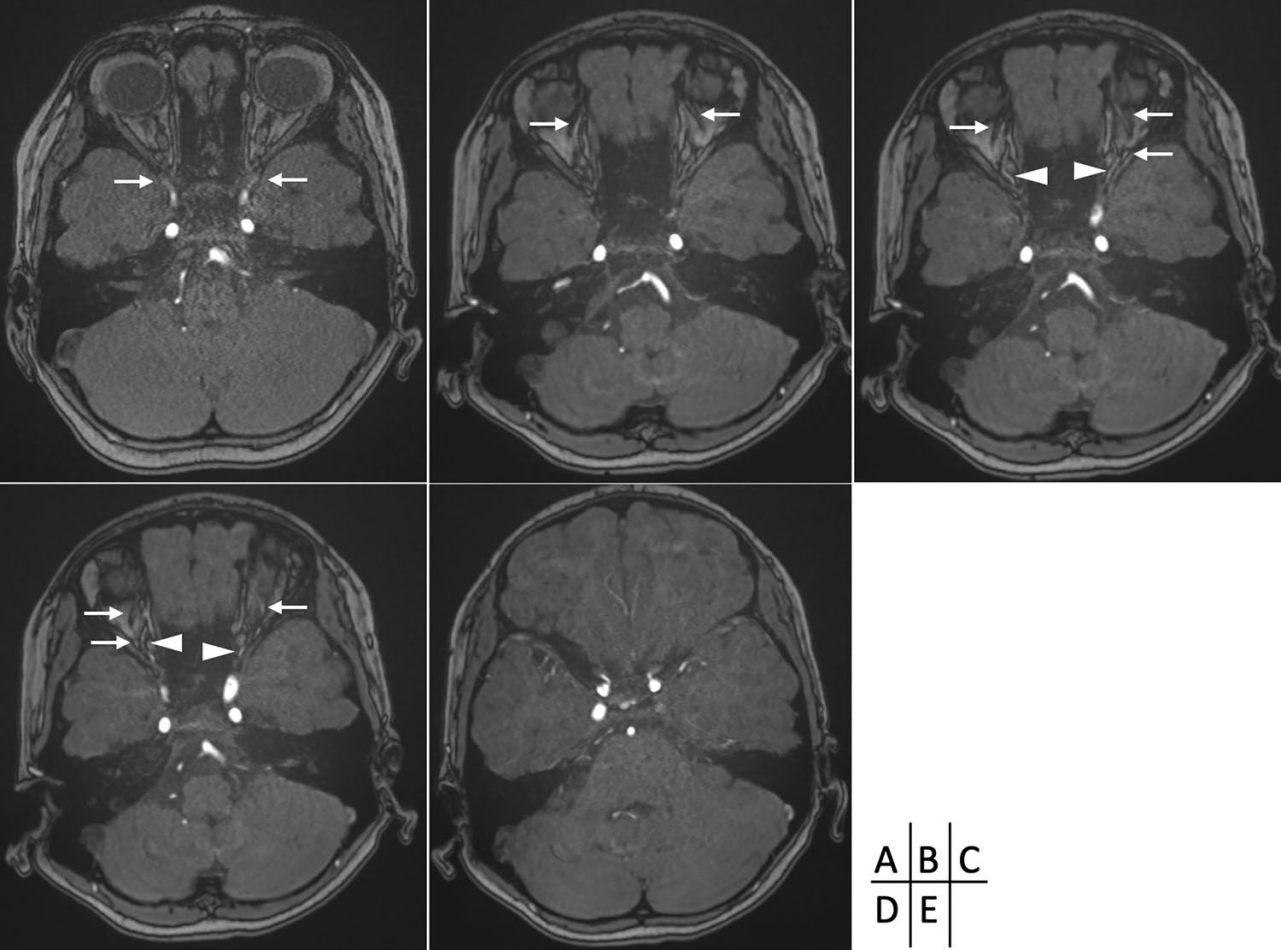
Six months after the initial magnetic resonance angiography (MRA), the origins of the bilateral primitive ophthalmic arteries (POAs) diminished. **A** Five years later, MRA revealed a natural regression of the POAs origins with a corresponding decrease in the POAs running within the orbit on bilateral sides. **B**, **C**, **D** No anastomosis was detected between the POAs and the ophthalmic artery (OA) within the orbit. **C**, **D** The OA was observed unchanged. **E** The arrows indicate the POAs. The arrowheads indicate the OA

Her headache, present at the initial consultation, improved. MRA were obtained again six months later and revealed decreased visualization of the origins of the POAs with visible terminals (Fig. 2). Five years later, MRA revealed disappearance of the origins of the bilateral POAs with diminished POAs running within the orbit and towards the terminals (Fig. 2). However, owing to the possibility of recanalization of the POAs in the future, we plan to continue with regular follow-up.

## Discussion

The Primitive ophthalmic artery (POA) is formed through a series of complex embryological processes [4, 8].

According to Padget's and Lasjaunias' hypotheses, the POA in this case is the dorsal ophthalmic artery (DOA) [4, 8]. In either hypothesis, the anastomosis, called the arterial ring, between the VOA and DOA may have been inadequate, suggesting that the POA persisted from birth in this case. It is thought that after birth, the ophthalmic artery that remained behind disappeared as the tissues were nourished by normal OA alone. Although there have been case reports of duplicate ophthalmic arteries, none that we have been able to locate indicate that the primitive ophthalmic arteries disappeared after birth [1–3, 5–7, 9, 10]. In this case, the primitive ophthalmic artery regressed naturally during follow-up, without any specific treatment or intervention.

In the first place, contrast-enhanced computed tomography (CT) or magnetic resonance imaging (MRI) are rarely performed in children unless shunting diseases such as dural arteriovenous fistula or neoplastic lesion is suspected. Additionally, it is suggested that in children, there may be instances of developmental immaturity, which raises the possibility that the natural regression of primitive ophthalmic arteries (POAs) may exist, even if it has not been confirmed through imaging or literature.

In any case, it might be helpful to be aware of the presence of a normal variant, as in the present case, to aid in proper diagnosis.

In this case, she was 2 years old at the time the primitive ophthalmic artery was noted on MRA. Therefore, it is a limitation of this case report that no imaging studies such as computed tomography angiography (CTA) or digital subtraction angiography (DSA) were performed due to the effects of radiation exposure.

## Conclusions

We encountered a case of regression of bilateral persistent primitive ophthalmic artery. No similar case has been reported in the relevant English-language literature [1–10].

## Data Availability

No datasets were generated or analysed during the current study.
